# Supplementary material for: Overexpression of Grapevine VvIAA18 Gene Enhanced Salt Tolerance in Tobacco
Source: Int J Mol Sci. 2020 Feb 15;21(4):1323. doi: 10.3390/ijms21041323 (PMC7072961; doi:10.3390/ijms21041323)
Supplement: Supplementary file 1 [file ijms-21-01323-s001.pdf]

FIGURES

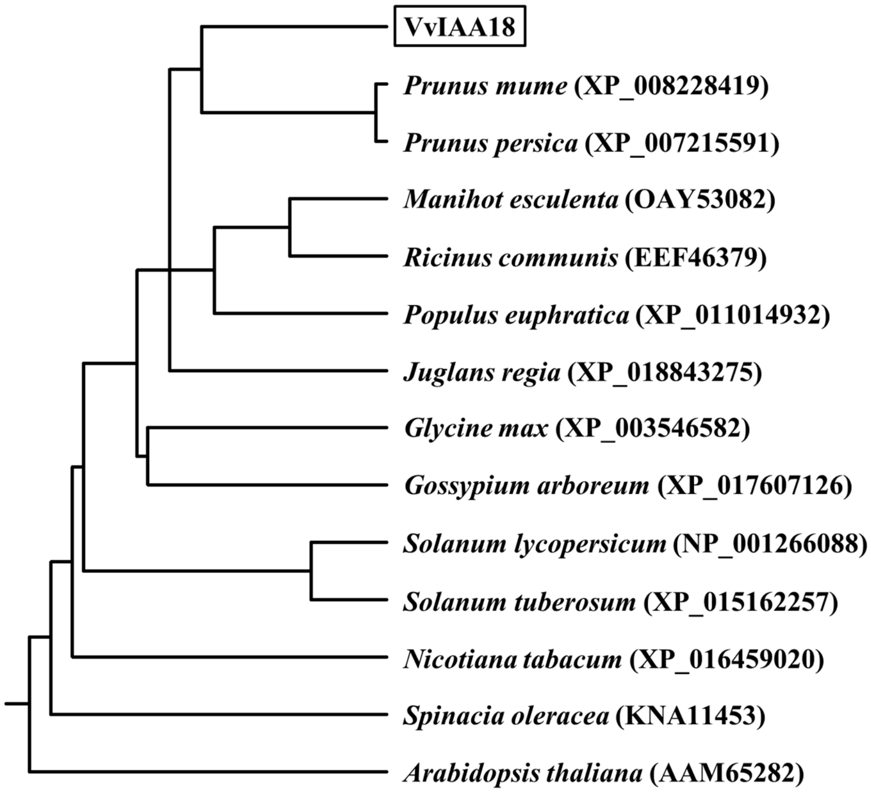

Figure 1. A phylogenetic tree of the VvIAA18 protein with its other plant homologous proteins. The branch lengths are proportional to the distance.

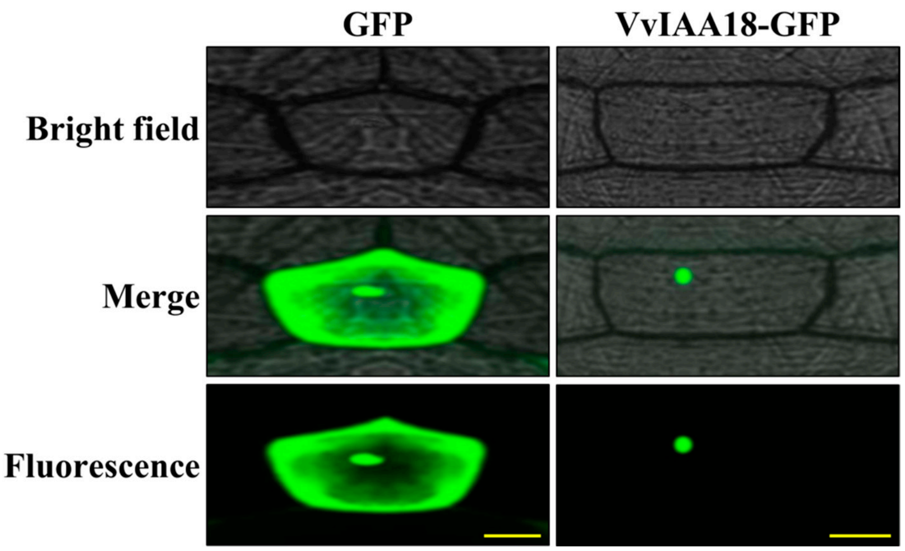

Figure 2. Subcellular localization of the VvIAA18 protein in onion epidermal cells. The VvIAA18-GFP fusion protein was localized to the nucleus. Scale bar = 100 μm.

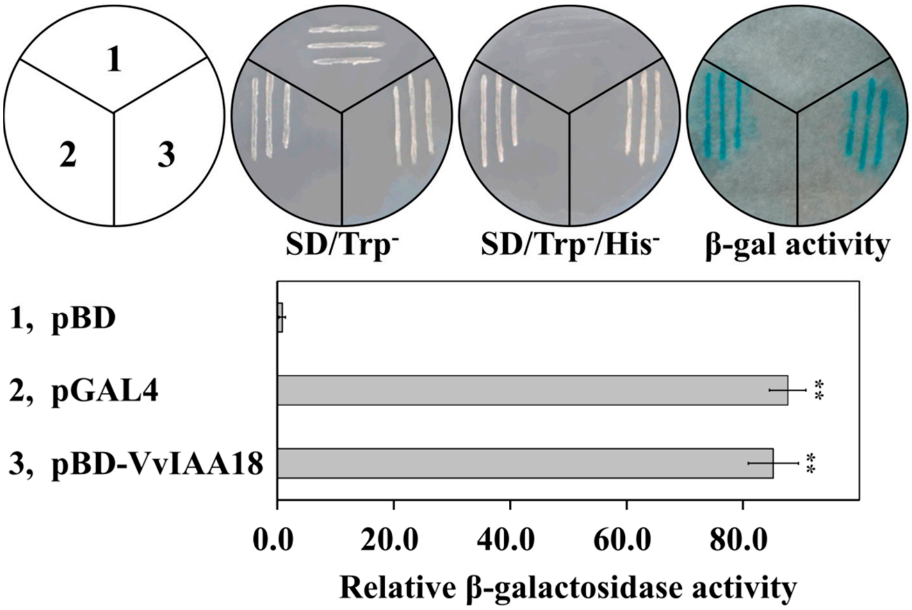

Figure 3. Transactivation assay of the VvIAA18 protein in the yeast. Fusion protein of the GAL4 DNA-binding domain and VvIAA18 were expressed in yeast. Use the empty pBD (pGBKT7) vector (negative control) and the pGAL4 vector (positive control). The culture solution of the transformed yeast was dropped onto SD plates without tryptophan or histidine. The plates were incubated for 3 days and then subjected to β-galactosidase assay.

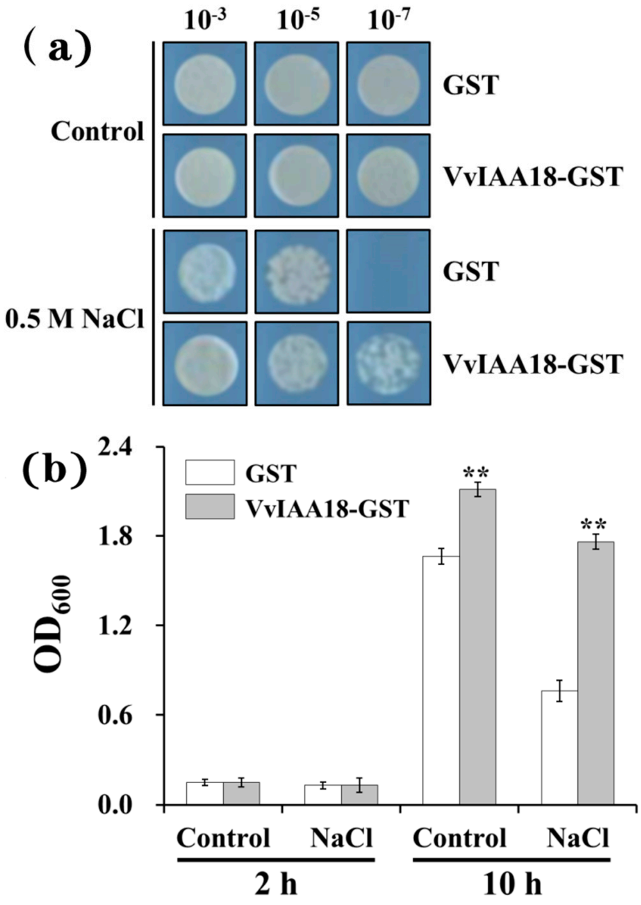

Figure 4. Enhanced salt tolerance in *Escherichia coli*. (**a**) Growth analysis of cells spotted on LB agar plate supplemented with 0.5 M NaCl. (**b**) Growth analysis of cells cultured in liquid medium supplemented with 0.5 M NaCl. Cell growth densities were measured at 600 nm at the indicated time points.

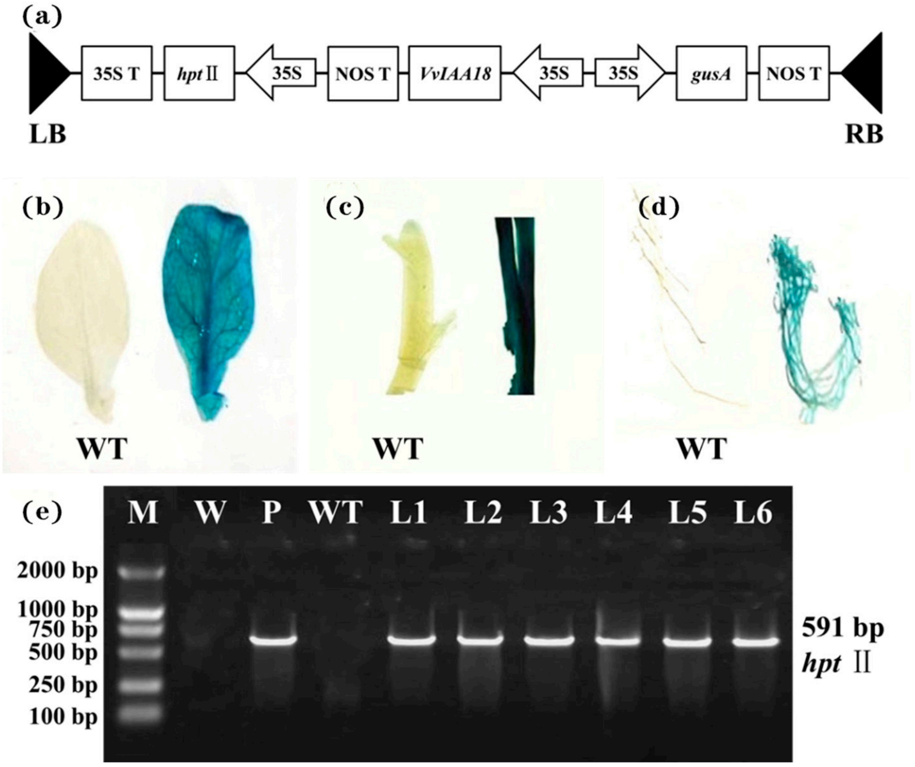

Figure 5. Molecular confirmation of transgenic plants. (**a**) Schematic diagram of the double plasmid pCAMBIA1301-*VvIAA18* T-DNA region. LB, left border; RB, right border; *hpt* II, hygromycin phosphotransferase II gene; *VvIAA18*, grape Aux/IAA transcription factor gene; *gusA*, β-glucuronidase gene; 35S, cauliflower mosaic virus (CaMV) 35S promoter; 35S T, CaMV 35S terminator; NOS T, nopaline synthase terminator. (**b**) (**c**) and (**d**) GUS expression in leaf, stem and root of a transgenic plant and no GUS expression in the wild-type (WT) (bar = 10 mm). (**e**) PCR analysis of *VvIAA18*-overexpressing tobacco plants. Lane M: DL2000 DNA marker; Lane W: water as negative control; Lane P: plasmid pCAMBIA1301-*VvIAA18* as positive control; Lane WT: wild type; Lanes L1-L6: different transgenic lines.

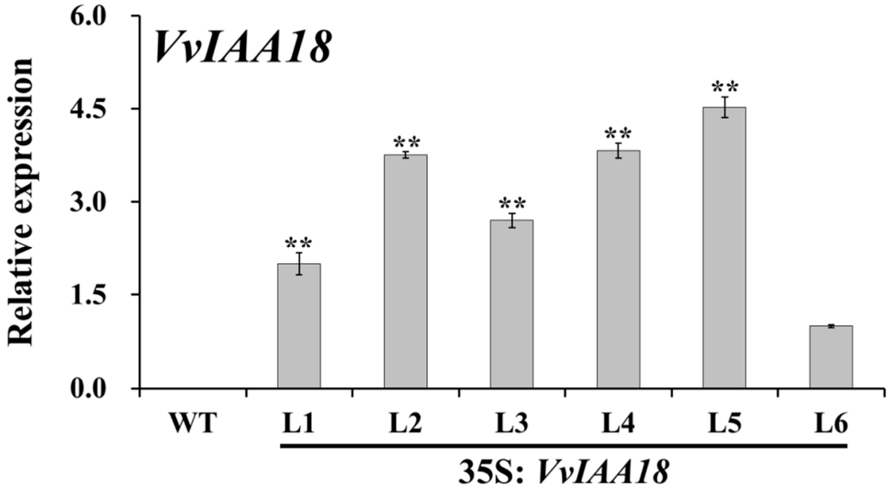

Figure 6. Real-time quantitative PCR expression analysis of VvIAA18 gene in transgenic tobacco plants. The expression of VvIAA18 in vitro-grown plants of 6 transgenic plants and wild type was analyzed. The tobacco Ntactin gene was used as an internal control. Data are presented as means ± SE (n=3). * And ** were significantly different from WT by $P < 0.05$ and $P < 0.01$ by Student's t-test, respectively.

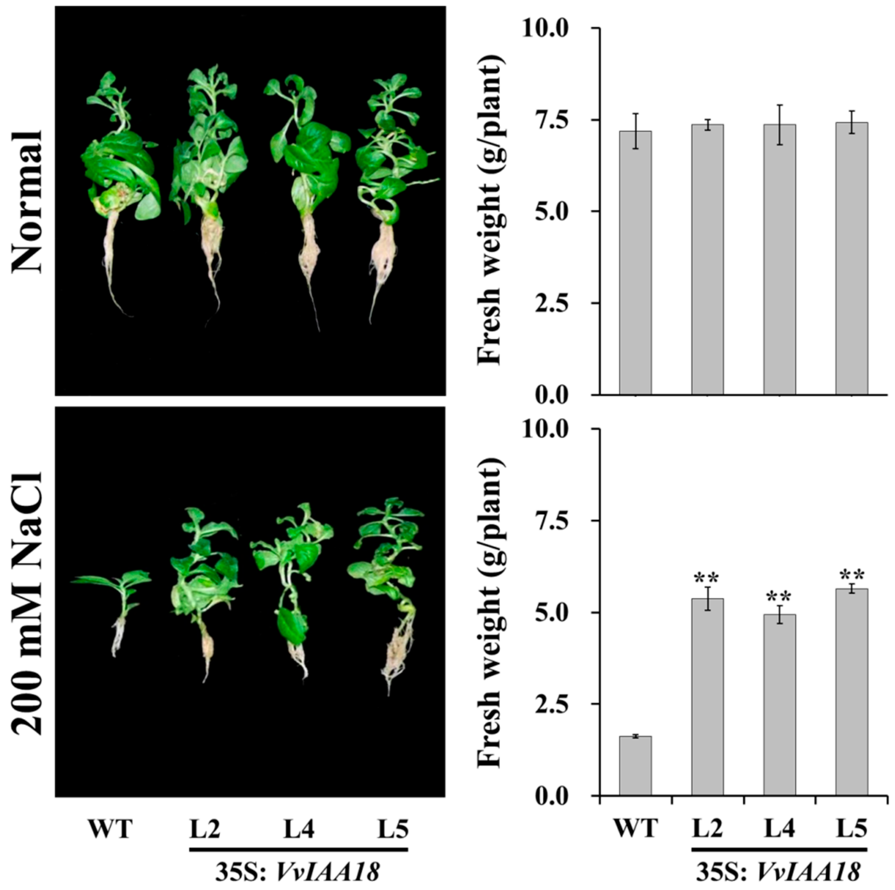

Figure7. The growth and rooting of transgenic plants were compared and WT was cultured for 4 weeks on MS medium with no stress or 200 mM NaCl. Data are presented as means ± SE (n=3). * And ** were significantly different from WT by P < 0.05 and P < 0.01 by Student's t-test, respectively.

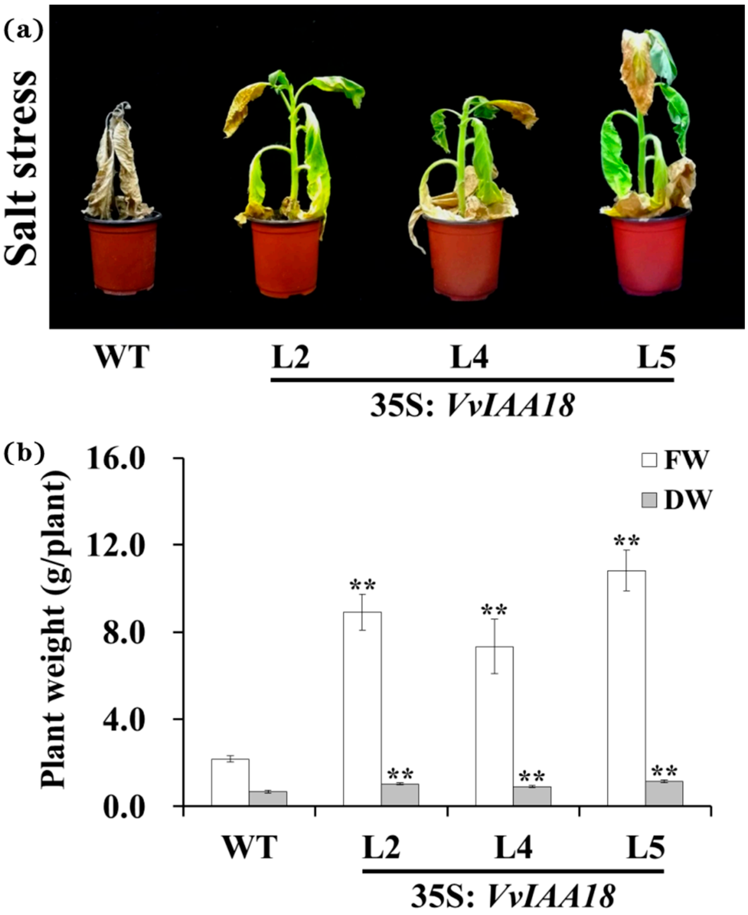

Figure 8. Responses of the transgenic tobacco plants and WT grown in pots under salt stress. (a), Phenotypes of the transgenic tobacco plants grown in pots under 200 mM NaCl stress. (b), Biomass of the transgenic tobacco plants grown in pots under 200 mM NaCl stress. Data are presented as means ± SE (n=3). * And ** were significantly different from WT by $P < 0.05$ and $P < 0.01$ by Student's *t*-test, respectively.

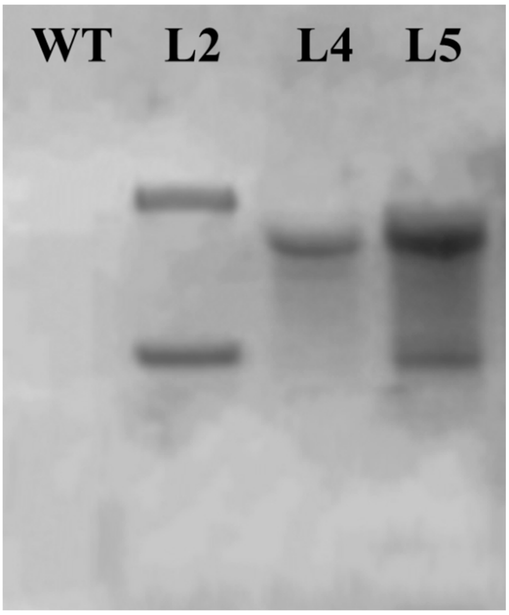

Figure 9. Southern blot analysis of the transgenic plants to detect the copy number of integrated *hpt*Ⅱ gene. WT, wild type; L1, L4 and L5, enhanced salt tolerance transgenic plants.

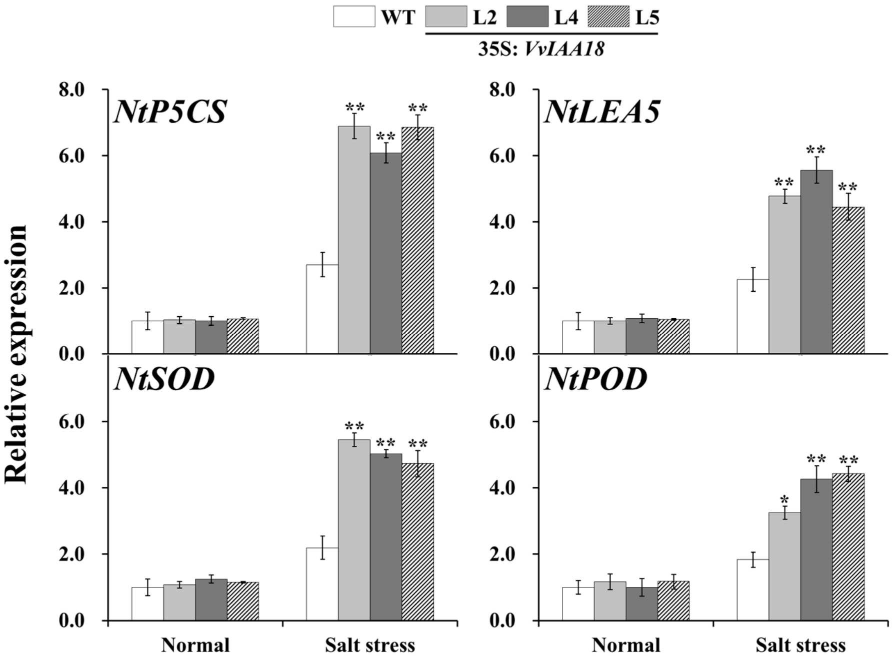

Figure 10. Relative expression level of salt stress-responsive genes in the leaves of transgenic tobacco plants and WT under salt stress. The tobacco *Ntactin* gene was used as an internal control. Results are expressed as relative values with respect to WT, which was set to 1.0. Data are presented as means ± SE (n=3). * And ** were significantly different from WT by $P < 0.05$ and $P < 0.01$ by Student's t-test, respectively.

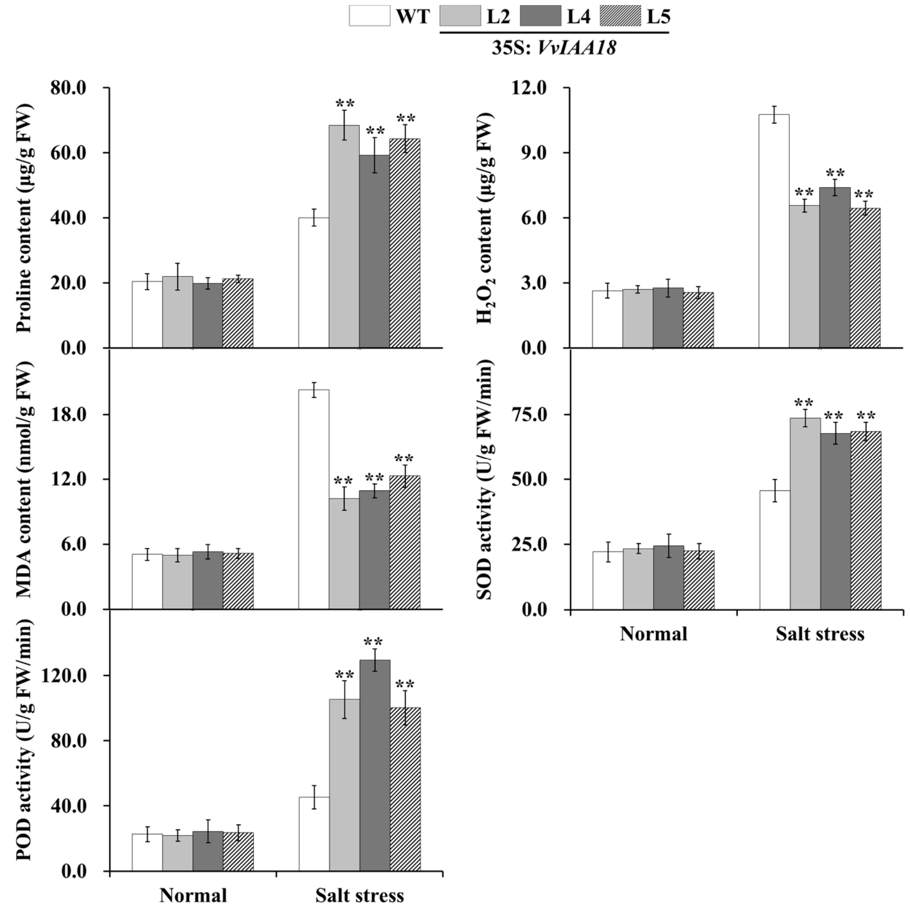

Figure 11. The content of proline, H_2O_2 and MDA, and the activities of SOD and POD in the leaves of transgenic tobacco plants and WT under salt stress. Data are presented as means ± SE (n=3). * And ** were significantly different from WT by $P < 0.05$ and $P < 0.01$ by Student's t-test, respectively.

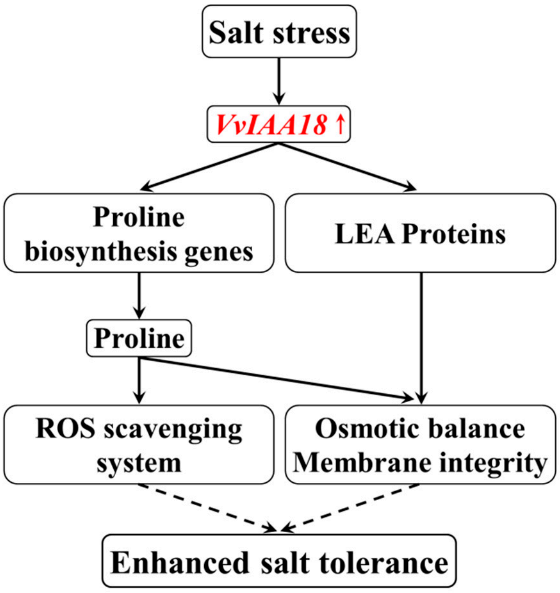

Figure 12. Hypothesis of the regulatory network of the *VvIAA18* gene involved in salt stress response. Constitutive expression of *VvIAA18* up-regulates the genes involved in proline biosynthesis and ROS scavenging, which result in significant physiological changes, including increased proline level and reduced ROS accumulation, leading to the enhanced salt tolerance.

Table s1 Primers used in this study.

Primer name	Primer sequence (5′-3′)
Primers for gene cloning for cDNA	
VvIAA18-GC-F	ATGGAGGGGTGTTCAAGGAAG
VvIAA18-GC-F	TCATTTCAATGCAGAGTCAAGTGG
Primers for subcellular localization of VvIAA18	
VvIAA18-SL-F	AAACTAGTATGGAGGGGTGTTCAAGGAA
VvIAA18-SL-F	AAGGCGCGCCTCATTTCAATGCAGAGTCAAGTG
Primers for transactivation assay of VvIAA18	
VvIAA18-TA-F	TGCATATGATGGAGGGGTGTTCAAGGAAGG
VvIAA18-TA-F	ACGGATCCTCATTTCAATGCAGAGTCAAGTGGT
Primers for constructing expression vector of VvIAA18	
VvIAA18-OE-F	CGGGATCCATGGAGGGGTGTTCAAGG
VvIAA18-OE-R	TTCGAGCTCTCATTTCAATGCAGAGTCAAG
Primers for identifying transformants	
VvIAA18-PCR-F	ACAGCGTCTCCGACCTGATGCA

VvIAA18-PCR-R	AGTCAATGACCGCTGTTATGCG
Primers for real-time quantitative PCR	
VvIAA18-F	CGAGTCCCAAGATGTGGTCC
VvIAA18-R	GCGTATATTCGCCACTCCCA
NtP5CS-F	CTGATGGAAGATTAGCACTTGG
NtP5CS-R	CTCCTACAGCACCTGAAGTC
NtLEA5-F	TTGAATCTGGGGTTTTGGTT
NtLEA5-R	GGAAGCATTGACGAGCTAGG
NtSOD-F	CTCCTACCGTCGCCAAAT
NtSOD-R	GCCCAACCAAGAGAACCC
NtPOD-F	CTTGGAACACGACGTTCCTT
NtPOD-R	TCGCTATCGCCATTCTTTCT
Ntactin-F	AGCAGCATGAAGATTAAGGTTGTAGCAC
Ntactin-R	TGGAAAATTAGAAGCACTTCCTGTGAAC

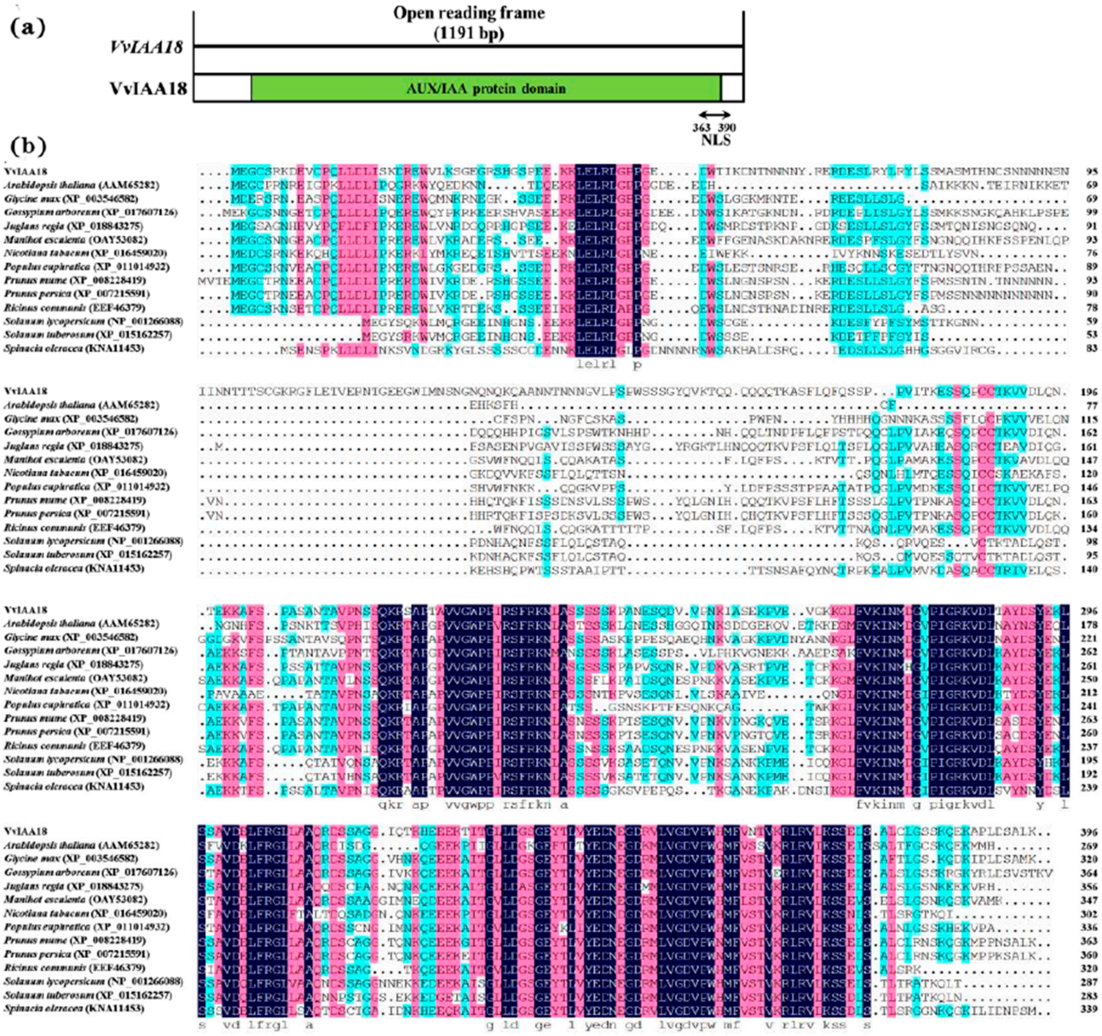

Figure s1 (a) Structure analysis of the VvIAA18 protein from grapevine. The VvIAA18 protein contains an AUX/IAA protein domain. **(b)** Sequence alignment of the VvIAA18 protein with its

homologous proteins from other plant species.
